# Sexual well-being in old age: are older adults well sexually?

**DOI:** 10.1080/07853890.2021.1896878

**Published:** 2021-09-28

**Authors:** Sofia von Humboldt

**Affiliations:** ISPA – Instituto Universitário, William James Center for Research, Lisbon, Portugal

## Abstract

**Introduction:**

Older adults who engage in sexual activities may benefit from increasing psychological and physical well-being, which may contribute to reduce a number of physical and mental health problems. The objectives of this study are the following: To analyse sexual well-being (SWB) in older adults’ perspective and to examine the potential explanatory mechanisms of a SWB overall model, in an older cross-national sample.

**Materials and methods:**

Measures were completed, using a variety of appropriate methods, including demographics and interviews. Complete data were available for 326 older adults aged between 65 and 102 years. Data were subjected to content analysis. Representation of the associations and latent constructs were analysed by a Multiple Correspondence Analysis (MCA).

**Results:**

The most prevalent response of the interviewed participants for SWB was “touching and caring” (18.0%). A three-dimension model formed by “care and well-being”, “attractiveness, intimacy and touching”, and “sexual intercourse and pleasure” was presented as a best-fit solution for English older adults. SWB for Portuguese older adults were explained by a three-factor model: “health and desire”, “care, eroticism and affection” and “penetration sex”.

**Discussion and Conclusions:**

The outcomes presented in this paper emphasised the need to explore the diversity of indicators of SWB among older adults and the cultural differences of a SWB model for older adults.

